# Infant Respiratory Outcomes Associated with Prenatal Exposure to Maternal 2009 A/H1N1 Influenza Vaccination

**DOI:** 10.1371/journal.pone.0160342

**Published:** 2016-08-03

**Authors:** Deshayne B. Fell, Kumanan Wilson, Robin Ducharme, Steven Hawken, Ann E. Sprague, Jeffrey C. Kwong, Graeme Smith, Shi Wu Wen, Mark C. Walker

**Affiliations:** 1 Better Outcomes Registry & Network (BORN) Ontario, Ottawa, Ontario, Canada; 2 Department of Medicine, University of Ottawa, Ottawa, Ontario, Canada; 3 Institute for Clinical Evaluative Sciences, Ottawa and Toronto, Ontario, Canada; 4 Clinical Epidemiology Program, Ottawa Hospital Research Institute, Ottawa, Ontario, Canada; 5 School of Epidemiology, Public Health and Preventive Medicine, University of Ottawa, Ottawa, Ontario, Canada; 6 Dalla Lana School of Public Health, University of Toronto, Toronto, Ontario, Canada; 7 Public Health Ontario, Toronto, Ontario, Canada; 8 Department of Family and Community Medicine, University of Toronto, Toronto, Ontario, Canada; 9 Department of Obstetrics & Gynaecology, Queen’s University, Kingston, Ontario, Canada; 10 OMNI Research Group, Department of Obstetrics and Gynecology, University of Ottawa, Ottawa, Ontario, Canada; 11 Department of Obstetrics, Gynecology and Newborn Care, The Ottawa Hospital, Ottawa, Ontario, Canada; US Food and Drug Administration, UNITED STATES

## Abstract

**Background:**

Infants are at high risk for influenza illness, but are ineligible for vaccination before 6 months. Transfer of maternal antibodies to the fetus has been demonstrated for 2009 A/H1N1 pandemic vaccines; however, clinical effectiveness is unknown. Our objective was to evaluate the association between 2009 A/H1N1 pandemic vaccination during pregnancy and rates of infant influenza and pneumonia.

**Methods:**

We linked a population-based birth cohort to administrative databases to measure rates of influenza and pneumonia diagnosed during ambulatory physician visits, hospitalizations and emergency department visits during one year of follow-up. We estimated incidence rate ratios and 95% confidence intervals (95% CI) using Poisson regression, comparing infants born to A/H1N1-vaccinated women (vaccine-exposed infants) with unexposed infants, adjusted for confounding using high-dimensional propensity scores.

**Results:**

Among 117,335 infants in the study, 36,033 (31%) were born to A/H1N1-vaccinated women. Crude rates of influenza during the pandemic (per 100,000 infant-days) for vaccine-exposed and unexposed infants were similar (2.19, 95% CI: 1.27–3.76 and 3.60, 95% CI: 2.51–5.14, respectively), as were crude rates of influenza and pneumonia combined. We did not observe any significant differences in rates of study outcomes between study groups during the second wave of the 2009 A/H1N1 pandemic, nor during any post-pandemic time period.

**Conclusion:**

We observed no difference in rates of study outcomes among infants born to A/H1N1-vaccinated mothers relative to unexposed infants born during the second A/H1N1 pandemic wave; however, due to late availability of the pandemic vaccine, the available follow-up time during the pandemic time period was very limited.

## Introduction

Pregnant women are considered a high-risk group for serious influenza illness and influenza-related complications. The World Health Organization [1] and many countries [2–5] advise vaccination of pregnant women with inactivated influenza vaccine in any trimester. Uptake of these recommendations has been increasing [6], particularly during the 2009 A/H1N1 influenza pandemic [7] when pregnant women were prioritized for pandemic vaccination programs and strongly encouraged to become immunized.

Aside from prevention of maternal influenza disease, increasing evidence supports that influenza immunization during pregnancy confers newborn seroprotection against influenza illness. This is clinically important since respiratory illness due to influenza is one of the most common reasons for hospitalizations of infants [8,9], yet those younger than 6 months are ineligible for influenza vaccination [10]. Transplacental transfer of maternal anti-influenza antibodies has been documented in immunogenicity studies of seasonal trivalent inactivated influenza vaccines (TIV) [11,12] as well as monovalent 2009 A/H1N1 pandemic vaccines [13,14]. Clinical efficacy of TIV administration during pregnancy on reducing infant influenza illness has been reported by three randomized controlled trials (RCTs) [11,12,15]; however, TIV effectiveness studies using observational designs have generated inconsistent results [16–22]. To our knowledge, only one small study has specifically assessed whether monovalent 2009 A/H1N1 pandemic vaccine administered to pregnant women was of further benefit to newborns during the 2009 A/H1N1 pandemic [23]. Our objective was to assess the effect of 2009 A/H1N1 pandemic vaccination in pregnancy on rates of infant influenza during one year of follow-up.

## Methods

### Study population and data sources

This retrospective cohort study included all hospital live births ≥500 grams or ≥20 weeks of gestation to Ontario residents between November 2, 2009 and October 31, 2010. This period corresponded with a one-year initiative to collect information on maternal influenza vaccination, concomitant with the availability of the monovalent A/H1N1 pandemic vaccine. We defined this cohort using maternal-newborn records from Better Outcomes Registry & Network (BORN) Ontario, a population-based birth registry that collects detailed clinical and demographic information on all births in the province.

We used deterministic and probabilistic methods to link the infant cohort with health administrative databases at the Institute for Clinical Evaluative Sciences (ICES) to ascertain influenza-coded health care encounters among infants (our proxy for clinical influenza illness). The ICES Registered Persons Database (RPDB) provided demographic information for the record linkage and information on eligibility for health care services during the follow-up period. Three health service databases identified health care visits for the specific clinical diagnoses that defined our study outcomes: the Canadian Institute for Health Information (CIHI) Discharge Abstract Database, containing information on hospital admissions; the CIHI National Ambulatory Care Reporting System, which provided information on ambulatory care received in hospitals (including emergency departments (ED)); and the Ontario Health Insurance Plan Claims Database, which recorded ambulatory office visits to physicians. Under the publicly-funded health care system, all Ontario residents have access to the services captured by these databases (further description provided in Text A in S1 File). The study population was restricted to records that were successfully linked to the RPDB, and those with complete information on maternal receipt of A/H1N1 pandemic vaccine during pregnancy (i.e., the study exposure).

### Follow-up and influenza time periods

**Fig 1:**
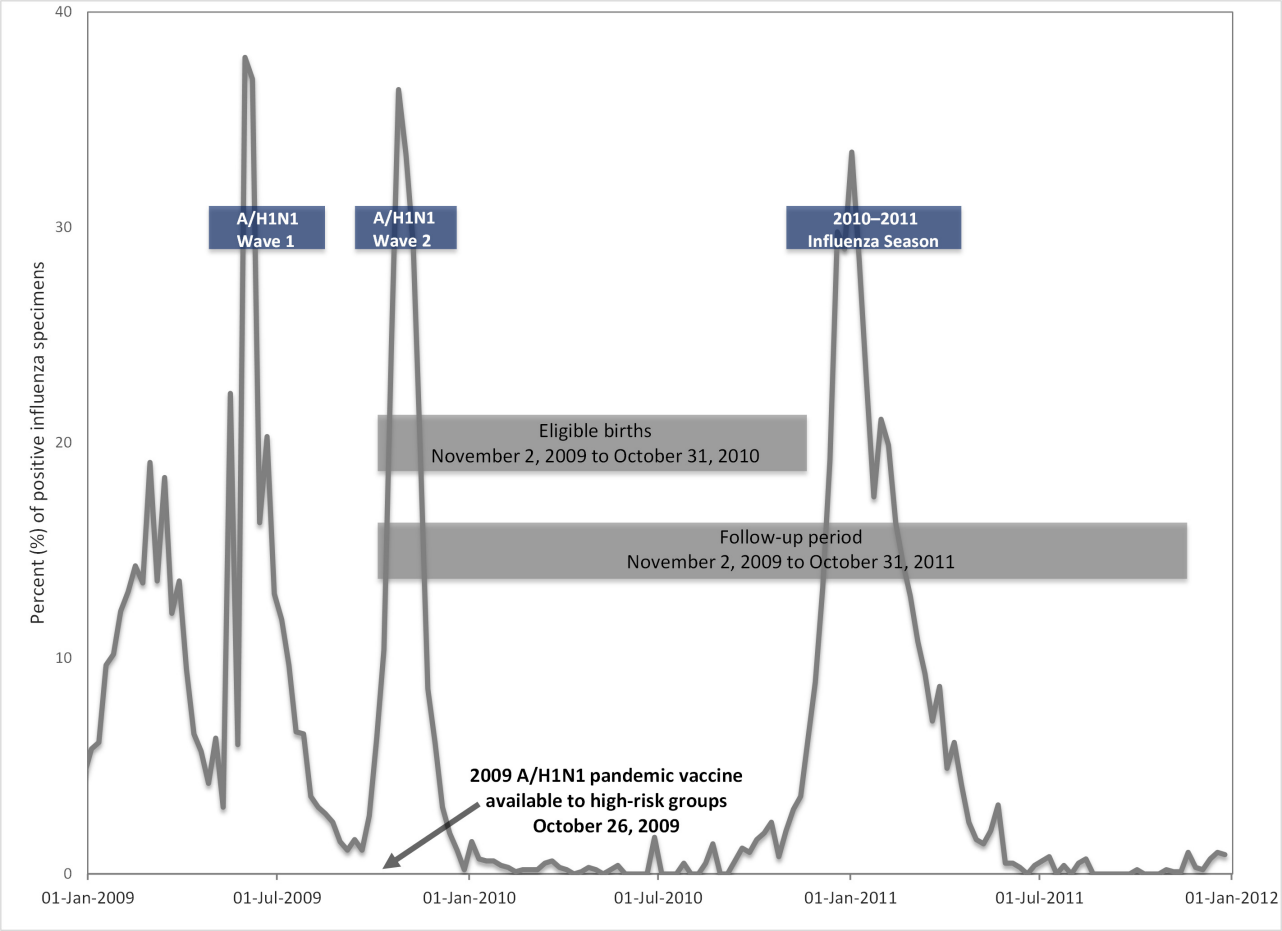
Eligible births and follow-up time in relation to influenza time periods.

Infants accrued days of follow-up beginning at birth and ending on their first birthday. In cases of emigration or death, follow-up only reflected the time during which they were eligible to receive health care services in Ontario. The A/H1N1 influenza pandemic encompassed two waves––the first beginning in April 2009, followed by a second beginning in September 2009, peaking in November 2009 and subsiding in late December 2009 [24]. The time period between the birth of the first infants in the cohort (November 2, 2009) and the end of follow-up for the youngest infants (October 31, 2011) traversed two influenza seasons: the second wave of the 2009 A/H1N1 pandemic, and the 2010–2011 influenza season (Fig 1) in which influenza A/H3N2 circulation predominated [25]. We used provincial virologic surveillance data to define the bounds of each influenza season. We considered the start of each season as the first occurrence of two consecutive weeks in which at least 5% of specimens submitted to provincial surveillance laboratories tested positive for influenza A or B viruses [26]. The end of each season was defined as the last occurrence of two consecutive weeks with ≥5% positive specimens; however, we extended the boundary for the second wave of the A/H1N1 pandemic in our analyses to February 6, 2010, since there continued to be case reporting in the province (personal communication: Public Health Ontario, August 2012). The time period between September 1st and the start of each influenza season was considered the pre-influenza time period and the time between the end of each season until August 31^st^ was considered the post-influenza season (Table 1) [27].

**Table 1:** Influenza time periods.

Time period	Start	Stop
2009 A/H1N1 pandemic	02 Nov 2009^a^	06 Feb 2010
Post 2009 A/H1N1 pandemic	07 Feb 2010	31 Aug 2010
Pre-2010-2011 season	01 Sept 2010	20 Nov 2010
2010–2011 season	21 Nov 2010	02 Apr 2011
Post-2010-2011 season	03 Apr 2011	31 Aug 2011
Pre-2011–2012 season	01 Sept 2011	31 Oct 2011^b^

^a^ Date corresponds to the first births in the study cohort

^b^ Date corresponds to the end of one-year of follow-up for the last births in the study cohort

### Measures

Using information from the birth registry, we classified infants born to mothers with documented 2009 A/H1N1 vaccination during pregnancy as vaccine-exposed, while infants born to mothers who were not vaccinated against A/H1N1 pandemic influenza during pregnancy were considered unexposed. The A/H1N1 pandemic influenza vaccination campaign in Ontario started on October 26, 2009, and most pregnant women received a non-adjuvanted vaccine.

Our primary outcome was infant influenza, defined as an influenza diagnostic code in a primary or a secondary field within any of the three health administrative databases (Table A in S1 File). When an infant had more than one health care visit with an influenza code, they were counted as unique events if the end of the first visit and the start of the next were more than seven days apart. Although the validity of influenza diagnostic codes in administrative health databases has not been established for infants, a validation study in the general Ontario population during the 2009 H1N1 pandemic time period compared laboratory data (gold standard) with health administrative data using the same diagnostic codes as we used in this study and found that sensitivity of influenza-coded health care visits was 28% in the physician billings database, 50% in the emergency department database and 76% in the hospitalization database, while corresponding specificity values were 85%, 84% and 95%, respectively (Kwong JC, unpublished data, 2015).

Given that outcomes coded as influenza in health administrative databases tend to underestimate the true incidence (i.e., low sensitivity) [28], in sensitivity analyses we also assessed a more sensitive, but less specific, outcome based on a combination of diagnostic codes for influenza or pneumonia (Table A in S1 File), since influenza virus infection can cause severe illness through different pathways including primary viral pneumonia and secondary pneumonia from bacteria or other pathogens [29,30]. We also examined patterns of ED visits and hospitalizations for any cause during follow-up as a surrogate marker for general differences in patterns of illness and/or health care seeking behaviour between the exposure groups.

### Statistical analyses

We described the study population using frequencies for categorical variables, and means and medians for continuous variables, and compared characteristics using standardized differences, where absolute differences >10% were considered indicative of imbalance between the two exposure groups [31]. Crude incidence rates per 100,000 infant-days of follow-up were calculated for each exposure group and outcome, within specific time periods defined by influenza activity.

Poisson regression models were used to generate incidence rate ratios (IRR) and 95% confidence intervals (95% CI), comparing rates of study outcomes among infants born to A/H1N1-vaccinated women with unexposed infants, within time periods defined by influenza activity including non-influenza time periods. Our *a priori* study hypothesis was that infants born to mothers who were vaccinated against A/H1N1 during their pregnancy would have lower rates of influenza-coded health care encounters during the defined H1N1 pandemic period compared with infants whose mothers did not receive the H1N1 vaccination. However, no difference was expected during non-influenza time periods nor during the subsequent influenza season (due to a lack of viral circulation or a mismatch between the pandemic vaccine and circulating virus in the subsequent season, respectively). Including non-influenza time periods has been recommended as one way of exposing possible bias due to preferential vaccine receipt by healthier individuals, a well-known phenomenon in observational studies of elderly influenza vaccine recipients [32].

We first performed unadjusted analyses to compare crude incidence rates followed by adjusted analyses using high-dimensional propensity score methods to account for confounding. We developed our propensity score model using logistic regression to estimate the probability of A/H1N1 pandemic influenza vaccination during pregnancy. The model included investigator-defined preselected covariates from the birth registry (e.g., maternal smoking, maternal age, multifetal gestation; full list provided in Table B in S1 File) as well as 500 covariates (from a pool of 8,703 potential covariates) selected from administrative databases using a previously-developed algorithm. The C-statistic for the high-dimensional propensity score model was 0.77. Propensity scores were categorized into deciles and included in all of our adjusted models. We used multiple imputation to impute missing values for the preselected demographic and clinical covariates from the birth registry prior to running the propensity score model. Five imputation cycles were carried out and the model results from each of the five imputed datasets were combined to account for the variation across multiply imputed values.

We used descriptive summary statistics and standardized differences to compare maternal and infant characteristics and rates of study outcomes among records with and without complete information on vaccination to assess whether missing exposure was associated with other factors that could impact the validity of our results. All analyses were carried out using SAS Version 9.3 (SAS Institute, Cary, NC) on a UNIX platform.

### Ethics Statement

This study was approved by the Research Ethics Boards of the Children’s Hospital of Eastern Ontario and the Ottawa Hospital Research Institute. According to Ontario provincial privacy legislation, informed consent is not required for studies using routinely collected data for secondary analysis. All study data were stored and analyzed in a secure network environment.

## Results

Of the 135,812 live birth records in the registry, 98.8% were linked to administrative databases. Following administrative exclusions (1,956 infants) and exclusions due to missing A/H1N1 vaccination information (14,941 infants), 117,335 infant records (representing 87% of all live birth records) were available for analysis (Fig A in S1 File). Only 3.3% of the total study follow-up time occurred during the A/H1N1 influenza pandemic time period (4.5% of the days of follow-up for infants born to A/H1N1-vaccinated mothers and 2.8% of non-exposed infant-days of follow-up; Fig B in S1 File). A total of 36,033 infants (31%) were exposed to maternal A/H1N1 pandemic vaccination while *in utero* (Table 2). Infants born to A/H1N1-vaccinated mothers were less likely to be in the lowest neighbourhood income quintile, less likely to be born to a woman younger than 25 years of age and more likely to be born earlier in the study period.

**Table 2:** Maternal and infant characteristics by exposure group.

Characteristic	Infant exposed to maternal 2009 A/H1N1 vaccination during pregnancy				Standardized difference ^a^
	Yes		No		
	n = 36,033		n = 81,302		
	n	%	n	%	
**Birth weight (grams)**					
<2,500	2,155	6.0	5,256	6.5	2.1
2,500–2,999	5,612	15.6	14,006	17.2	4.3
3,000–3,499	13,362	37.1	30,527	37.5	0.8
3,500–3,999	10,836	30.1	23,150	28.5	3.5
≥4,000	4,068	11.3	8,363	10.3	3.2
**Gestational age (completed weeks)**					
<32	326	0.9	840	1.0	1.0
32–33	341	0.9	731	0.9	0
34–36	2,049	5.7	4,672	5.7	0
≥37	33,317	92.5	75,059	92.3	0.8
**Infant sex**					
Male	18,651	51.8	41,889	51.5	0.6
Female	17,379	48.2	39,406	48.5	0.6
Missing	<6	--	7	0	--
**Maternal medical co-morbidity ^**b**^**					
Yes	3,044	8.4	5,193	6.4	7.7
No	31,930	88.6	74,100	91.1	8.3
Missing	1,059	2.9	2,009	2.5	2.5
**Neighbourhood income quintile**					
1 (lowest)	6,503	18.0	19,734	24.3	15.5
2	6,465	17.9	16,709	20.6	6.9
3	7,183	19.9	16,282	20.0	0.3
4	8,345	23.2	16,577	20.4	6.8
5 (highest)	7,293	20.2	11,342	14.0	16.5
Missing	244	0.7	658	0.8	1.2
**Type of birth**					
Vaginal	25,026	69.5	58,344	71.8	5.1
Cesarean	10,985	30.5	22,897	28.2	5.1
Missing	22	0.1	61	0.1	0.3
**Maternal age (years)**					
<20	841	2.3	3,143	3.9	9.2
20–24	3,295	9.1	11,829	14.5	16.8
25–34	22,910	63.6	49,734	61.2	5.0
35–39	7,432	20.6	13,552	16.7	10.0
≥40	1,555	4.3	3,044	3.7	3.1
**Month of delivery**					
November 2009	3,241	9.0	5,513	6.8	8.2
December 2009	4,261	11.8	4,845	6.0	20.5
January 2010	4,599	12.8	5,003	6.2	22.7
February 2010	4,072	11.3	4,740	5.8	19.8
March 2010	4,144	11.5	5,974	7.3	14.4
April 2010	3,532	9.8	6,170	7.6	7.8
May 2010	3,234	9.0	6,994	8.6	1.4
June 2010	2,530	7.0	7,586	9.3	8.4
July 2010	2,220	6.2	7,891	9.7	13.0
August 2010	1,828	5.1	8,462	10.4	19.9
September 2010	1,442	4.0	8,969	11.0	26.8
October 2010	930	2.6	9,155	11.3	34.7
**Multiple gestation**					
Yes	1,404	3.9	2,629	3.2	3.8
No	34,629	96.1	78,673	96.8	3.8
**Pregnancy induced hypertension or pre-eclampsia**					
Yes	1,994	5.5	3,917	4.8	3.2
No	33,344	92.5	76,440	94.0	6.0
Missing	695	1.9	945	1.2	5.7
**Rural residence**					
Yes	4,864	13.5	9,357	11.5	6.1
No	31,165	86.5	71,930	88.5	6.1
Missing	<6	--	15	0.0	--
**Smoking during pregnancy**					
Yes	3,259	9.0	9,538	11.7	8.9
No	31,185	86.5	68,751	84.6	5.4
Missing	1,589	4.4	3,013	3.7	3.6

^a^ Expressed as an absolute percentage

^b^ Asthma, chronic hypertension, insulin dependent diabetes, non-insulin dependent diabetes or heart disease

**Fig 2:**
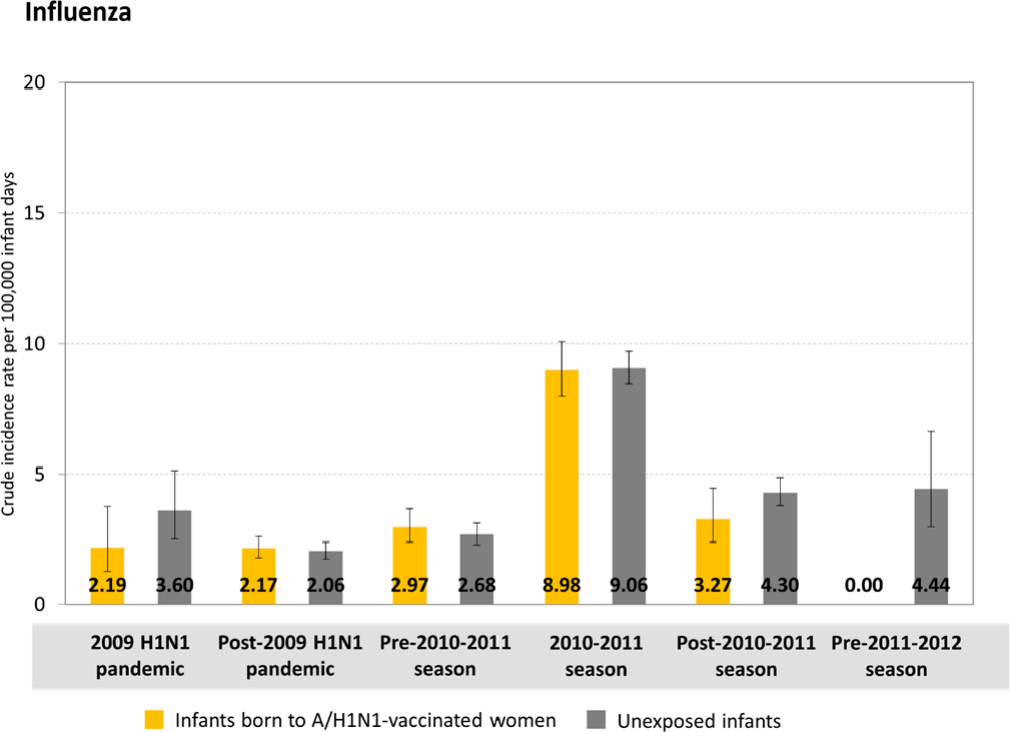
Crude incidence rates (per 100,000 infant-days of follow up) of influenza-coded health care encounters by exposure group and influenza time period. Vertical bars denote 95% confidence intervals.

Crude incidence rates of influenza illness per 100,000 infant-days of follow-up during the A/H1N1 pandemic time period were low and did not differ meaningfully between vaccine-exposed and unexposed infants (2.19, 95% CI: 1.27–3.76 and 3.60, 95% CI: 2.51–5.14, respectively; Fig 2). During the same time period, crude incidence rates of influenza and pneumonia combined were higher in magnitude but also did not differ between vaccine-exposed and unexposed infants (Fig C in S1 File). Compared with unexposed infants, A/H1N1 pandemic vaccination during pregnancy was not associated with any reduction in influenza and pneumonia combined during the pandemic A/H1N1 time period (adjusted IRR: 1.04, 95% CI: 0.84–1.29; Table C in S1 File). We were unable to generate a stable adjusted estimate for influenza alone during the pandemic time period due to the low number of outcomes; however, the crude IRR was not statistically significant (unadjusted IRR: 0.61, 95% CI: 0.32–1.17) and unadjusted and adjusted estimates in other time periods did not differ meaningfully from each other in magnitude nor direction (Table 3). We observed a similar pattern of null results for influenza (Fig 2 and Table 3) and for influenza and pneumonia combined (Fig C in S1 File and Table C in S1 File) in all other post-pandemic time periods, including the 2010–2011 influenza season. There was no indication of any overall difference in patterns of all-cause ED visits or hospitalizations among infants in any follow-up time period (Fig D in S1 File).

**Table 3:** Unadjusted and adjusted incidence rate ratios (IRR), 95% confidence intervals (CI) for influenza-coded health care encounters, comparing infants born to A/H1N1-vaccinated mothers with unexposed infants by influenza time period.

Influenza time period	Influenza	
	Unadjusted	Adjusted
	IRR, 95% CI	IRR, 95% CI^a^
2009 A/H1N1 pandemic ^b^	0.61, 0.32–1.17	*Indeterminate* ^*c*^
Post 2009 A/H1N1 pandemic	1.06, 0.83–1.34	1.05, 0.81–1.37
Pre-2010-2011 season	1.11, 0.85–1.44	1.08, 0.80–1.44
2010–2011 season	0.99, 0.87–1.13	0.88, 0.76–1.02
Post-2010-2011 season	0.76, 0.54–1.07	0.72, 0.50–1.04
Pre-2011–2012 season	*Indeterminate* ^*c*^	*Indeterminate* ^*c*^

^a^ Adjusted using high-dimensional propensity scores

^b^ Second wave of the 2009 A/H1N1 pandemic only

^c^ A stable estimate during this time period could not be generated due to the low number of outcomes

We compared characteristics of records with and without complete information on A/H1N1 pandemic influenza vaccination during pregnancy and found no differences in crude rates of study outcomes, nor in the distribution of birth weight, gestational age at birth, month of birth, plurality, or maternal age (Table D in S1 File). The proportion of infants whose mother had a pre-existing medical comorbidity, developed pre-eclampsia during pregnancy or smoked during pregnancy did not differ; however, the records missing vaccination information also had more missing values for these three variables.

## Discussion

In this population-based cohort study, we examined the association between monovalent A/H1N1 pandemic influenza vaccination during pregnancy and rates of influenza-coded health care encounters over a one-year follow-up period (our proxy for clinical infant influenza disease). We observed no reduction in rates of influenza-coded health care visits among vaccine-exposed infants during the latter part of the second A/H1N1 pandemic wave; however, the available follow-up time during the defined A/H1N1 pandemic time period was very limited. As expected, we similarly did not observe any differences in rates of influenza-coded health care encounters among infants born to A/H1N1-vaccinated mothers relative to unexposed infants during any post-pandemic time period, including the 2010–2011 influenza season when influenza A/H3N2 predominated.

In a real-world, non-RCT setting, the effectiveness of influenza vaccination in pregnancy as a strategy for infant protection depends on the timing of vaccine administration, the timing of influenza circulation, and the dynamic population of pregnant women and infants at risk for influenza [33,34]. In a temperate climate where influenza typically circulates for a limited period of two to three months each year, infants are most likely to benefit when influenza immunization of pregnant women occurs prior to, or early during an influenza season, and in later stages of gestation since the birth will more likely occur during the period of high viral circulation [33,35]. The second pandemic wave in Ontario had reached its peak by the time the A/H1N1 monovalent pandemic vaccine was distributed to pregnant women [35], leaving little time during which a direct benefit of the vaccine to newborns could be realized. The unpredictability of pandemics and possible vaccine delays represent an ongoing challenge for pandemic preparedness, as illustrated in this study.

The literature on influenza vaccination during pregnancy and infant respiratory outcomes is limited to a small number of studies. The highest quality evidence is provided by three RCTs conducted in low-resource settings, all consistently reporting significant reductions in laboratory-confirmed influenza among infants during six months of follow-up (by 46% among non-HIV infected participants in South Africa, by 63% in Bangladesh, and by 33% in Mali) [11,12,15]. A fourth RCT of TIV from Nepal is soon expected to add to this evidence [36]. In contrast, observational studies of TIV administration during pregnancy and infant influenza have yielded inconsistent findings possibly due to methodological issues such as small sample sizes [17–20,37], pooled data across influenza seasons with varying vaccine effectiveness [16–18,20–22], minimal adjustment for confounding variables [17,18,37], and reliance on clinical influenza definitions (e.g., medical visits with a diagnostic code for influenza recorded in a health administrative database) rather than laboratory-confirmed influenza [16,17,21]. Three observational studies that exclusively used clinical definitions found no association between TIV immunization during pregnancy and infant influenza [16,17,21]. Among four observational studies of laboratory-confirmed influenza, TIV effectiveness estimates ranged from 41% to 71% for infant influenza infection [19,37] and from 39% to 92% for infant influenza hospitalization [18–20,37]. One recent study pooled across nine influenza seasons and included both a clinical definition (influenza-like illness based on diagnostic codes in a health administrative database) as well as laboratory-confirmed influenza and found highly protective risk ratios for both (indicating a 66% reduction in infant influenza among those whose mothers were vaccinated during pregnancy), despite the clinically-defined influenza outcome being less specific and, therefore, being expected to yield a more conservative estimate of lower magnitude than the laboratory-confirmed influenza outcome [22]. Moreover, the investigators did not stratify their results by influenza season nor assess any of the study outcomes according to the unique timing of influenza circulation, which could have helped rule out the possibility that treatment selection bias was exaggerating the magnitude of their findings, particularly for the clinically-defined influenza outcome. To our knowledge, only one other study has specifically assessed pandemic A/H1N1 vaccination of pregnant women and subsequent infant influenza illness. Van der Maas and colleagues followed a cohort of Dutch infants from birth to one year of age and found no difference in rates of infection-related physician visits (adjusted incidence rate ratio 1.07; 95% CI: 0.91–1.28) between infants of unvaccinated and A/H1N1-vaccinated mothers. However, this study also did not account for the timing of viral circulation (pandemic A/H1N1 vaccine should only prevent influenza and related complications when the virus was circulating) and is further limited by a small sample size (fewer than 2,000 subjects), low questionnaire response rate (21%), and non-specific outcome (physician visits for any type of infection) [23].

Although RCTs are superior to observational designs for evaluating interventions such as influenza immunization, the former have had limited application in the obstetrical population due to ethical considerations that preclude randomization of pregnant women in settings where influenza immunization is the standard of care during pregnancy [10]. The RCTs of influenza immunization during pregnancy to-date have been small, conducted in low-resource settings, recruited mainly low-risk women in their second or third trimester, and have followed infants for safety outcomes only up to 6 months of age. Given these characteristics, the annual reformulation of influenza vaccines [5], and the unclear external validity of the RCT findings in high-resource settings and in climates with different temporal patterns of influenza circulation [5], observational studies will likely continue to play a role in the ongoing assessment of influenza vaccination during pregnancy under real-world conditions. However, there are a number of challenges faced by such studies, particularly when utilizing health administrative databases. These include a lack of validation studies for the diagnostic codes used to define study outcomes [38], a paucity of comprehensive data sources for information on vaccination during pregnancy (such as a registry), and insufficient information on potential confounding factors that can be used in statistical analyses to address bias due to preferential vaccine receipt by women with a more favourable risk profile [39]. This bias, which consistently operates in the direction of making influenza vaccination appear strongly protective against many health outcomes, is a well-described problem in observational studies of influenza vaccine in the elderly population [32,40,39]. The degree to which this “healthy vaccinee” effect operates in the much younger, healthier obstetrical population is unclear, but significantly reduced risk ratios for outcomes not known to be strongly associated with influenza disease (e.g., preterm birth) reported by some observational vaccination studies [41] may implicate analogous issues.

Our study differs from previous research in several ways. Whereas most aforementioned studies used some form of sampling and examined TIVs (many by combining data across several seasons with different TIV formulations), we studied a population-based cohort of live births, almost a third of whom were uniquely exposed to a monovalent A/H1N1 pandemic influenza vaccine while *in utero*. Although the temporal dynamics of the pandemic and the vaccine delay [35] constrained the available follow-up time during the defined A/H1N1 pandemic time period (when a vaccine effect could plausibly be expected), a distinguishing feature of our study was the length of infant follow-up (one year), which traversed a second influenza season with different viral characteristics (when a vaccine effect would not be expected). We are not aware of any other studies that have examined influenza vaccination during pregnancy and infant influenza illness in a subsequent season. Among adults, residual effectiveness of the 2009 monovalent pandemic vaccine in the 2010–2011 influenza season was demonstrated in settings where pandemic A/H1N1 continued to circulate [42], but not where the A/H3N2 viral subtype predominated [43]. Our findings agree with the latter study insofar as no beneficial effect of passively-acquired immunity to pandemic A/H1N1 influenza was seen in the A/H3N2-dominated 2010–2011 season. However, approximately two-thirds of infants in our study would have surpassed the age of four months by the time the 2010–2011 season began, making it unlikely that they would still have had adequate seroprotection even if the circulating virus matched the vaccine administered during pregnancy [12,44].

Studies in adult populations have recently drawn attention to the complex relationship between prior influenza vaccination and immune responses in a subsequent influenza season [45,46]. A series of studies during the 2009 pandemic found that receipt of the prior season’s TIV increased the risk of pandemic influenza illness [47], and other recent vaccination studies have noted higher vaccine effectiveness in the current year among participants who were not vaccinated in the prior season [48,49]. Given that pregnant women and infants aged six months to five years are currently prioritized for influenza immunization [1], infant immune systems will potentially be exposed to up to two (usually different) influenza vaccine formulations between conception and reaching their first birthday. While maternally-derived infant immunity to influenza is expected to wane during the postnatal period [12,44], possible residual effects from antenatal exposure of the fetal immune system to influenza vaccine on future immunological responses [45] have not been assessed and represent an important direction for future studies of influenza vaccination during pregnancy [50].

Our findings should be interpreted in consideration of the strengths and limitations of our study. A major strength was the availability of a population-based birth registry linked with administrative databases, enabling us to follow-up a large cohort of infants during a time period when maternal influenza vaccination rates were particularly high [7]. We expect that any misclassification of maternal A/H1N1 pandemic vaccination was likely non-differential with respect to our study outcome, which would have biased our estimates toward the null value. We used high-dimensional propensity score methods to adjust for confounding, an approach that reduces bias more effectively than conventional adjustment methods in studies using large administrative databases and efficiently controls for a large number of covariates within a single score.

The most important limitation affecting our study concerns the pandemic vaccine availability in relation to circulating A/H1N1 influenza [35], discussed earlier. The number of infants in our cohort born during the second pandemic wave after the vaccine became available to pregnant women was low, resulting in limited follow-up time and statistical power to detect differences between exposure groups. Another important limitation concerns misclassification of influenza due to the use of diagnostic codes in administrative databases as a proxy for clinical illness. We believe that both sensitivity and specificity of influenza diagnostic codes are likely higher in our study population than in the general population (see results from an Ontario validation study in the Methods), since young infants with any symptoms of influenza-like illness are more consistently brought to medical attention and laboratory testing of hospitalized infants is more routinely carried out than for hospitalized adults [51]. Nevertheless, since non-differential misclassification of our study outcome due to less than perfect sensitivity and specificity would be expected to have the net effect of biasing the IRR toward the null value, it follows that we could have underestimated a protective effect of maternal vaccination against infant A/H1N1 influenza during the pandemic. When the outcome is uncommon, as in our study, the magnitude of any bias introduced by non-differential outcome misclassification tends to be most impacted by low specificity [52], not by low sensitivity. Since we estimate the specificity in our study to be high, we expect any bias toward the null was relatively minor. Finally, while we excluded 11% of infant records from the analyses due to missing information on maternal influenza vaccination, we did not find any clinically meaningful differences between records with and without complete exposure information.

## Conclusion

In summary, during the second wave of the 2009 A/H1N1 influenza pandemic, we did not observe any reduction in rates of influenza or pneumonia among infants born to mothers who had received the monovalent pandemic vaccine during pregnancy compared with non-exposed infants. The limited infant follow-up time during the pandemic time period due to delayed availability of the pandemic vaccine illustrates the challenges of influenza immunization of pregnant women as a strategy to protect infants during a pandemic.

## Supporting Information

S1 File Text A. Description of databases and linkage methodology. Table A. Diagnostic codes used to identify infant respiratory outcomes in administrative databases. Table B. List of preselected demographic and clinical variables from the birth registry included in high-dimensional propensity score model. Fig A. Study flow diagram. Fig B. Distribution of study follow-up time by exposure group and influenza time period. Fig C. Crude incidence rates of influenza and pneumonia by exposure group and influenza time period. Fig D. Crude incidence rates of all-cause emergency department visits and hospitalizations by exposure group and influenza time period. Table C. Unadjusted and adjusted incidence rate ratios (IRR), 95% confidence intervals (CI) for influenza and pneumonia, comparing infants born to A/H1N1-vaccinated mothers with unexposed infants by influenza time period. Table D. Comparison of infant records with complete and incomplete information on A/H1N1 pandemic influenza vaccination during pregnancy.
(DOCX)

## Abbreviations

TIV: trivalent inactivated influenza vaccines
RCTs: randomized controlled trials
ICES: Institute for Clinical Evaluative Sciences
RPDB: Registered Persons Database
CIHI: Canadian Institute for Health Information
ED: emergency department
IRR: incidence rate ratio
95% CI: 95% confidence intervals
