# Supplementary material for: Infant Respiratory Outcomes Associated with Prenatal Exposure to Maternal 2009 A/H1N1 Influenza Vaccination
Source: PLoS One. 2016 Aug 3;11(8):e0160342. doi: 10.1371/journal.pone.0160342 (PMC4972313; doi:10.1371/journal.pone.0160342)
Supplement: S1 File — Text A. Description of databases and linkage methodology. Table A. Diagnostic codes used to identify infant respiratory outcomes in administrative databases. Table B. List of preselected demographic and clinical variables from the birth registry included in high-dimensional propensity score model. Fig A. Study flow diagram. Fig B. Distribution of study follow-up time by exposure group and influenza time period. Fig C. Crude incidence rates of influenza and pneumonia by exposure group and influenza time period. Fig D. Crude incidence rates of all-cause emergency department visits and hospitalizations by exposure group and influenza time period. Table C. Unadjusted and adjusted incidence rate ratios (IRR), 95% confidence intervals (CI) for influenza and pneumonia, comparing infants born to A/H1N1-vaccinated mothers with unexposed infants by influenza time period. Table D. Comparison of infant records with complete and incomplete information on A/H1N1 pandemic influenza vaccination during pregnancy. (DOCX) [file pone.0160342.s001.docx]

**Infant respiratory outcomes associated with prenatal exposure to maternal 2009 A/H1N1 influenza vaccination**

**S1 File – Supporting Information**

[Text A. Description of databases and linkage methodology 2](#_Toc456936798)

[Table A. Diagnostic codes used to identify infant respiratory outcomes in administrative databases 5](#_Toc456936799)

[Table B. List of preselected demographic and clinical variables from the birth registry included in high-dimensional propensity score model 6](#_Toc456936800)

[Fig A. Study flow diagram 7](#_Toc456936801)

[Fig B. Distribution of study follow-up time by exposure group and influenza time period 8](#_Toc456936802)

[Fig C. Crude incidence rates of influenza and pneumonia by exposure group and influenza time period 9](#_Toc456936803)

[Fig D. Crude incidence rates of all-cause emergency department visits and hospitalizations by exposure group and influenza time period 10](#_Toc456936804)

[Table C. Unadjusted and adjusted incidence rate ratios (IRR), 95% confidence intervals (CI) for influenza and pneumonia, comparing infants born to A/H1N1-vaccinated mothers with unexposed infants by influenza time period 11](#_Toc456936805)

[Table D. Comparison of infant records with complete and incomplete information on A/H1N1 pandemic influenza vaccination during pregnancy 12](#_Toc456936806)

[References 15](#_Toc456936807)

Text A. Description of databases and linkage methodology

**Better Outcomes Registry & Network (BORN) Ontario Birth Registry**

The Better Outcomes Registry & Network (BORN) Ontario birth registry captures all hospital births >500 grams or >20 weeks’ gestation in the province of Ontario. The routine data collection includes information on maternal demographic variables; pre-existing maternal health problems; obstetric factors affecting the index pregnancy; birth outcomes; and commencing November 2, 2009 for a one-year period, maternal A/H1N1 pandemic vaccination status. Data collected from medical records, clinical forms and patient interviews are entered in the database around the time when a woman is admitted to hospital to give birth. An ongoing program of data verifications, quality checks and formal training sessions for individuals collecting and entering data assures a high level of data quality is maintained.[1]

**Institute for Clinical Evaluative Sciences (ICES) Registered Persons Database**

The Institute for Clinical Evaluative Sciences (ICES) Registered Persons Database (RPDB) is a registration file containing basic demographic information for anyone who has received an Ontario health card number, including the time period for which each individual was eligible to receive publicly-funded health care in Ontario. This database contains the ICES Key Number (IKN), which is an encrypted identification variable enabling linkage between all administrative databases within ICES. The RPDB was used to assist with the linkage of the BORN birth registry to ICES databases.

**Canadian Institute for Health Information (CIHI) Discharge Abstract Database**

The Discharge Abstract Database (DAD) is a national database, compiled and administered by the Canadian Institute for Health Information (CIHI). The DAD includes hospital separation abstracts submitted by all acute care hospital facilities in Canada (excluding Quebec). On an annual basis, a file of Ontario hospital separations is transferred from CIHI to ICES for provincial use. The DAD contains demographic information, medical diagnoses (most responsible diagnosis and up to 24 secondary diagnoses), interventions received, length of hospital stay, vital disposition at time of discharge and other data elements. Medical diagnoses are coded using the Canadian implementation of the International Classification of Diseases, 10th Revision (ICD–10–CA).[2,3]

**Canadian Institute for Health Information (CIHI) National Ambulatory Care Reporting System**

The National Ambulatory Care Reporting System (NACRS) is also a national database administered by CIHI, with annual transfer of Ontario records to ICES. NACRS captures data originating from urgent visits to emergency departments in Ontario. Up to 10 clinical diagnoses on each abstract are coded using ICD–10–CA.[2]

**Ontario Health Insurance Plan (OHIP) Claims Database**

The Ontario Health Insurance Plan (OHIP) Claims Database records claims reimbursed by OHIP, which covers all health care providers permitted to submit claims under OHIP (including physicians, groups, laboratories, and out-of-province providers). Only one diagnosis (i.e., the underlying reason for the medical visit) is coded on each claim.

**Record linkage**

Linkage of the BORN birth registry data with ICES databases was carried out by the ICES Programming and Biostatistics Analyst following the secure transfer of an encrypted file containing the BORN data. The record linkage employed both deterministic and probabilistic methodologies using attributes from the combined maternal-newborn records in the birth registry. These attributes included hospital of birth and hospital chart numbers, maternal and newborn dates of birth, postal code of residence, infant sex, birth weight and gestational age. Following linkage to the RPDB, the anonymous ICES Key Number (IKN) was appended to birth registry records, enabling linkage with all other ICES administrative databases required for this study.

Table A. Diagnostic codes used to identify infant respiratory outcomes in administrative databases

| Study outcome | DAD | NACRS | OHIP |
| --- | --- | --- | --- |
|  | ICD–10–CA Codes ^a^ | ICD–10–CA Codes ^a^ | Diagnosis Codes ^b^ |
| Influenza (primary outcome) | J09–J11^c^ | J09–J11^c^ | 487 |
| Pneumonia and influenza | J09–J11^c^ | J09–J11^c^ | 487 |
|  | J12.0, J12.3, J12.8, J12.9 | J12.0, J12.3, J12.8, J12.9 | 480–486 |
|  | J13–J18 | J13–J18 |  |

^a^ Canadian implementation of the International Classification of Diseases, 10th Revision

^b^ For ambulatory physician visits, if a fee service code for influenza immunization was assigned on the same record as an influenza diagnostic code, the visit was not counted as a study outcome

^c^ During the 2009 pandemic, there was a directive to use the ICD-10-CA “J09” code for patients admitted to hospital with a laboratory-confirmed diagnosis of A/H1N1 infection or with high clinical suspicion [4]

DAD: Discharge Abstract Database; NACRS: National Ambulatory Care Reporting System; OHIP: Ontario Health Insurance Plan database

Table B. List of preselected demographic and clinical variables from the birth registry included in high-dimensional propensity score model

| Variable |
| --- |
| Gestational age |
| Infant sex |
| Maternal medical co-morbidity ^a^ |
| Neighbourhood income quintile |
| Maternal age |
| Nulliparity |
| Month of delivery |
| Multiple gestation |
| Previous preterm birth |
| Pregnancy induced hypertension or pre-eclampsia |
| Health region of residence |
| Rural residence |
| Smoking during pregnancy |

^a^ Asthma, chronic hypertension, insulin dependent diabetes, non-insulin dependent diabetes or heart disease

Fig A. Study flow diagram


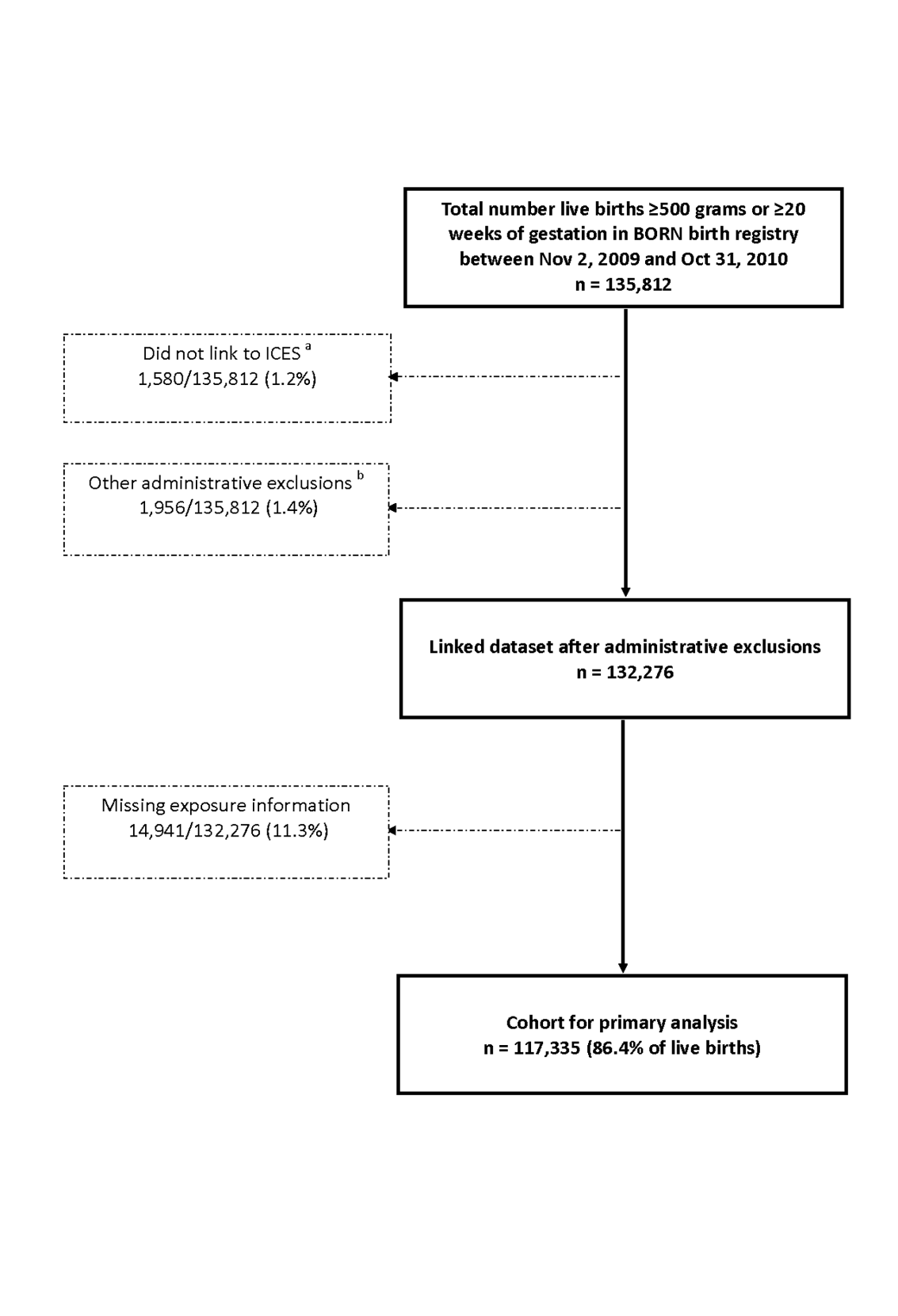


^a^ Neither the infant nor the mother linked to the ICES registration database

^b^ Administrative exclusions: no infant identified in ICES registration database (only the mother was linked) n=567; no infant health care claims in any of the databases and/or no valid infant sex or birth date information in ICES registration database (n=1,208); data cleaning (n=181)

BORN: Better Outcomes Registry & Network; ICES: Institute for Clinical Evaluative Sciences

Fig B. Distribution of study follow-up time by exposure group and influenza time period


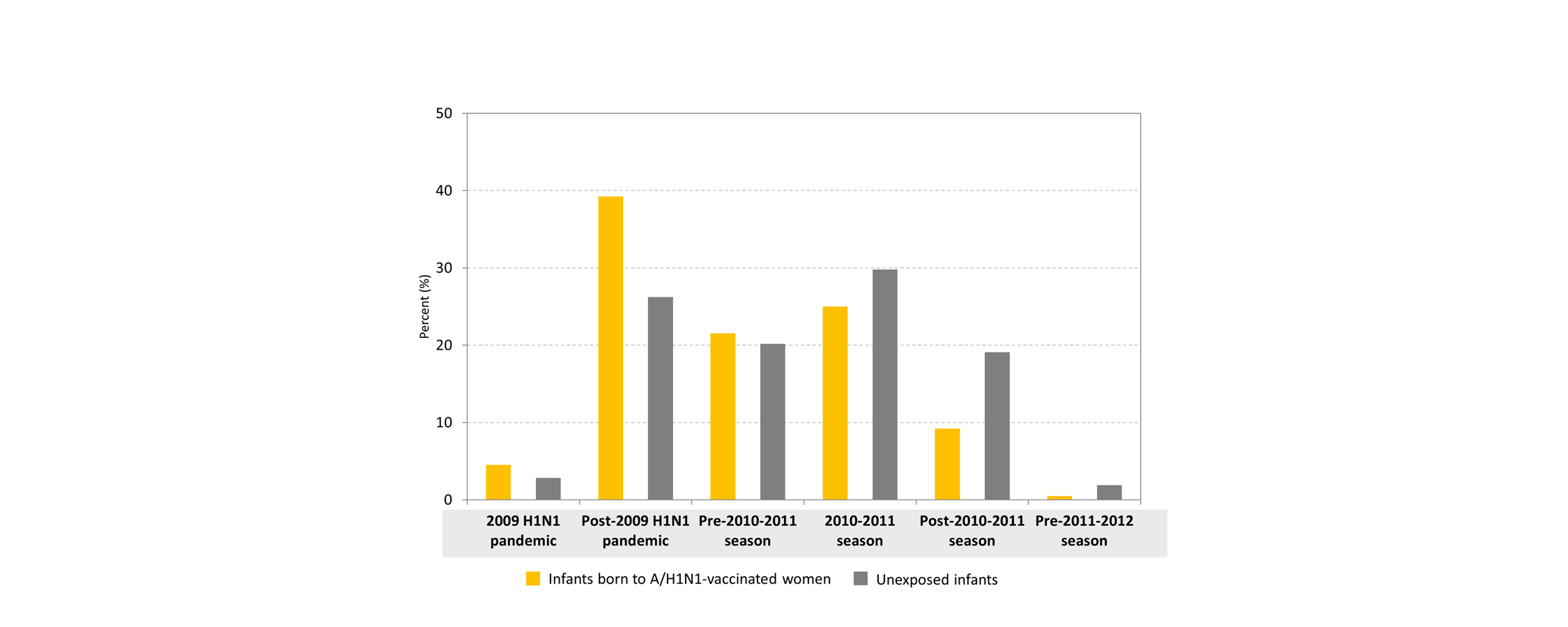


Fig C. Crude incidence rates of influenza and pneumonia by exposure group and influenza time period


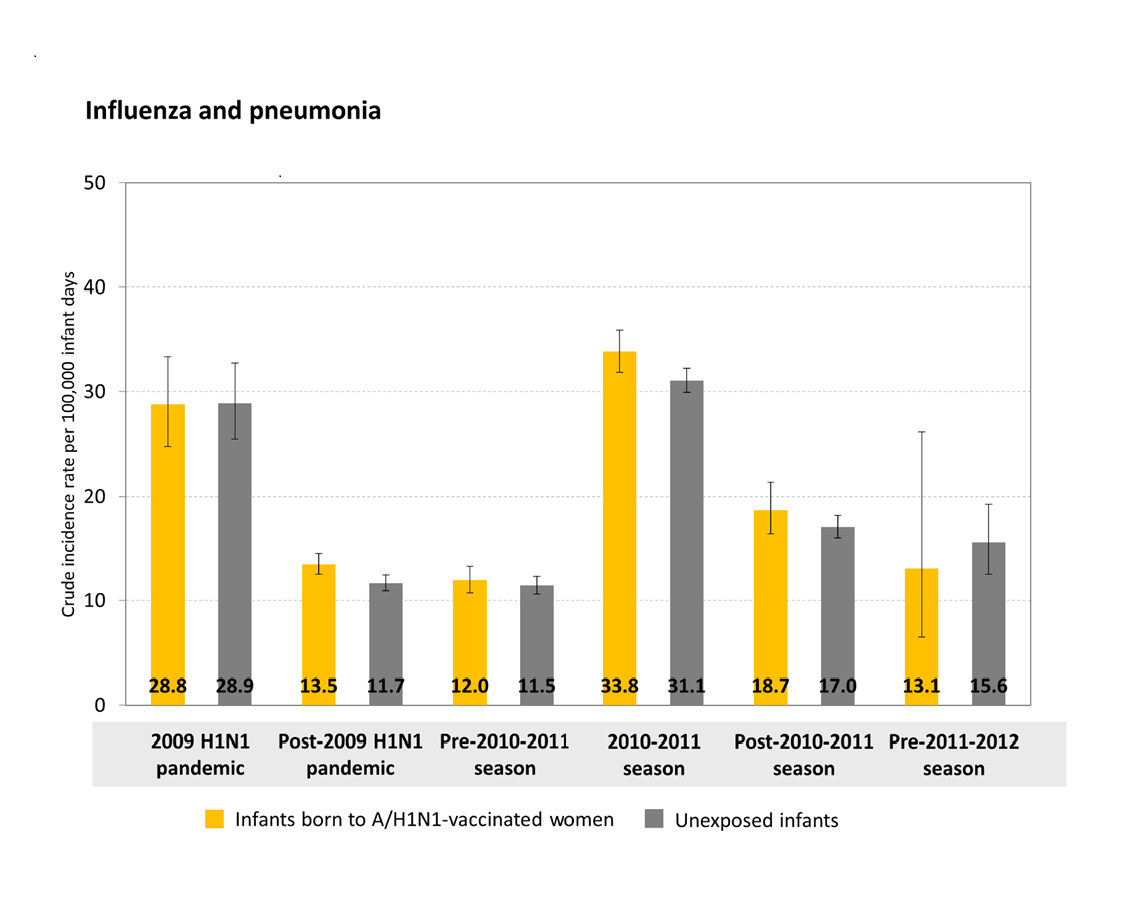


Fig D. Crude incidence rates of all-cause emergency department visits and hospitalizations by exposure group and influenza time period


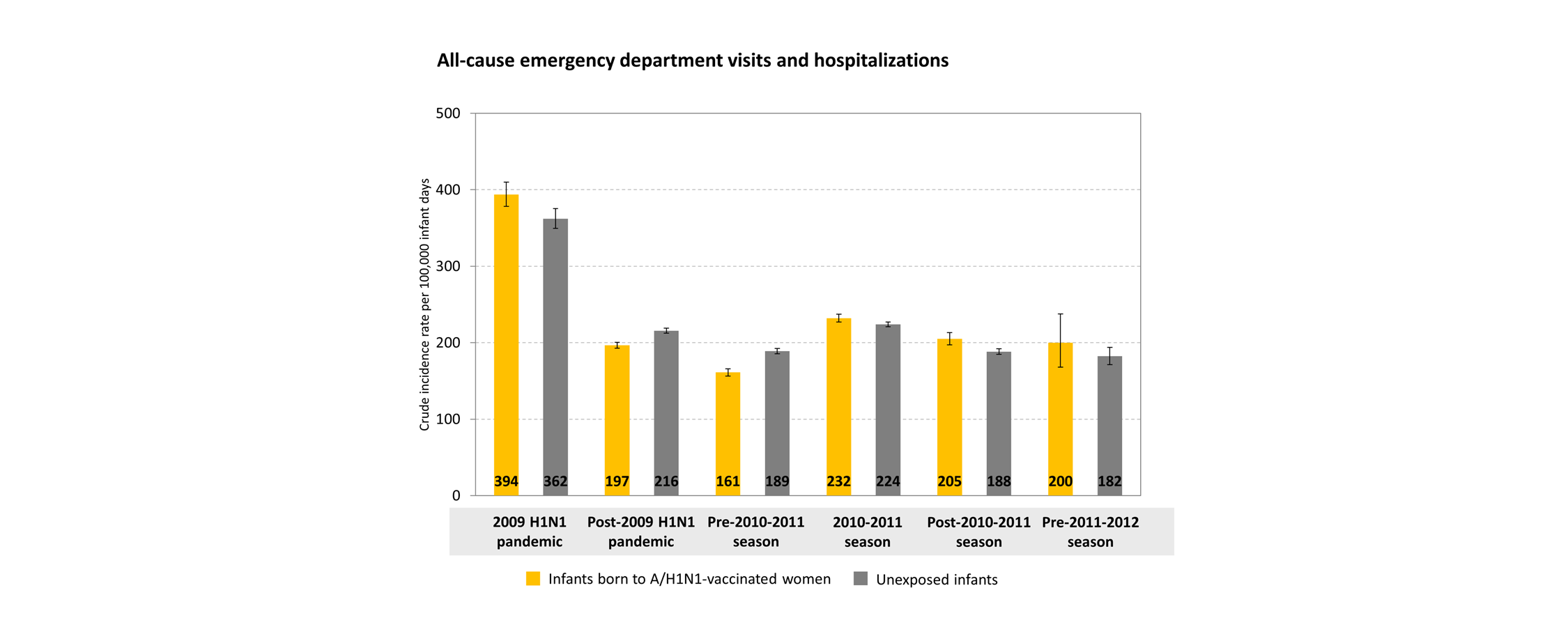


Table C. Unadjusted and adjusted incidence rate ratios (IRR), 95% confidence intervals (CI) for influenza and pneumonia, comparing infants born to A/H1N1-vaccinated mothers with unexposed infants by influenza time period

| **Influenza time period** | **Influenza and pneumonia** | |
| --- | --- | --- |
|  | **Unadjusted** | **Adjusted** |
|  | **IRR, 95% CI** | **IRR, 95% CI^a^** |
| 2009 A/H1N1 pandemic ^b^ | 1.00, 0.82–1.21 | 1.04, 0.84–1.29 |
| Post 2009 A/H1N1 pandemic | 1.16, 1.05–1.28 | 1.17, 1.05–1.31 |
| Pre-2010-2011 season | 1.04, 0.92–1.19 | 1.07, 0.93–1.25 |
| 2010-2011 season | 1.09, 1.02–1.17 | 0.99, 0.92–1.07 |
| Post-2010-2011 season | 1.10, 0.95–1.27 | 1.00, 0.85–1.17 |
| Pre-2011–2012 season | 0.84, 0.41–1.74 | 0.81, 0.38–1.70 |

^a^ Adjusted using high-dimensional propensity scores

^b^ Second wave of the 2009 A/H1N1 pandemic only

Table D. Comparison of infant records with complete and incomplete information on A/H1N1 pandemic influenza vaccination during pregnancy

| **Characteristic** | **Complete information on A/H1N1 pandemic influenza vaccination during pregnancy** | | **Standardized difference ^a^** |
| --- | --- | --- | --- |
|  | **No** | **Yes** |  |
|  | **n = 14,941** | **n = 117,335** |  |
|  | **n (%)** | **n (%)** |  |
| **Birth weight (grams)** |  |  |  |
| <2,500 | 1,030 (6.9) | 7,411 (6.3) | 2.4 |
| 2,500–2,999 | 2,537 (17.0) | 19,618 (16.7) | 0.8 |
| 3,000–3,499 | 5,503 (36.8) | 43,889 (37.4) | 1.2 |
| 3,500–3,999 | 4,256 (28.5) | 33,986 (29.0) | 1.1 |
| ≥4,000 | 1,615 (10.8) | 12,431 (10.6) | 0.7 |
| **Gestational age (completed weeks)** |  |  |  |
| <32 | 185 (1.2) | 1,166 (1.0) | 1.9 |
| 32–33 | 151 (1.0) | 1,072 (0.9) | 1 |
| 34–36 | 903 (6.0) | 6,721 (5.7) | 1.3 |
| ≥37 | 13,702 (91.7) | 108,376 (92.4) | 2.6 |
| **Maternal medical co-morbidity ^b^** |  |  |  |
| Yes | 933 (6.2) | 8,237 (7.0) | 3.2 |
| No | 12,725 (85.2) | 106,030 (90.4) | 15.9 |
| Missing | 1,283 (8.6) | 3,068 (2.6) | 26.3 |
| **Neighbourhood income quintile** |  |  |  |
| 1 (lowest) | 3,403 (22.8) | 26,237 (22.4) | 1 |
| 2 | 2,959 (19.8) | 23,174 (19.8) | 0 |
| 3 | 2,863 (19.2) | 23,463 (20.0) | 2 |
| 4 | 2,966 (19.9) | 24,924 (21.2) | 3.2 |
| 5 (highest) | 2,563 (17.2) | 18,635 (15.9) | 3.5 |
| Missing | 187 (1.3) | 902 (0.8) | 4.9 |
| **Maternal age (years)** |  |  |  |
| <20 | 555 (3.7) | 3,984 (3.4) | 1.6 |
| 20–24 | 1,954 (13.1) | 15,124 (12.9) | 0.6 |
| 25–34 | 9,135 (61.1) | 72,644 (61.9 | 1.6 |
| 35–39 | 2,719 (18.2) | 20,984 (17.9) | 0.8 |
| ≥40 | 578 (3.9) | 4,599 (3.9) | 0 |
| **Month of delivery** |  |  |  |
| November 2009 | 1,439 (9.6) | 8,754 (7.5) | 7.5 |
| December 2009 | 1,246 (8.3) | 9,106 (7.8) | 1.8 |
| January 2010 | 1,197 (8.0) | 9,602 (8.2) | 0.7 |
| February 2010 | 1,005 (6.7) | 8,812 (7.5) | 3.1 |
| March 2010 | 1,242 (8.3) | 10,118 (8.6) | 1.1 |
| April 2010 | 1,283 (8.6) | 9,702 (8.3) | 1.1 |
| May 2010 | 1,151 (7.7) | 10,228 (8.7) | 3.7 |
| June 2010 | 1,126 (7.5) | 10,116 (8.6) | 4 |
| July 2010 | 1,235 (8.3) | 10,111 (8.6) | 1.1 |
| August 2010 | 1,400 (9.4) | 10,290 (8.8) | 2.1 |
| September 2010 | 1,262 (8.4) | 10,411 (8.9) | 1.8 |
| October 2010 | 1,355 (9.1) | 10,085 (8.6) | 1.8 |
| **Multiple gestation** |  |  |  |
| Yes | 556 (3.7) | 4,033 (3.4) | 1.6 |
| No | 14,385 (96.3) | 113,302 (96.6) | 1.6 |
| **Pregnancy induced hypertension or pre-eclampsia** |  |  |  |
| Yes | 618 (4.1) | 5,911 (5.0) | 4.3 |
| No | 13,115 (87.8) | 109,784 (93.6) | 20.1 |
| Missing | 1,208 (8.1) | 1,640 (1.4) | 31.9 |
| **Smoking during pregnancy** |  |  |  |
| Yes | 1,529 (10.2) | 12,797 (10.9) | 2.3 |
| No | 11,283 (75.5) | 99,936 (85.2) | 24.6 |
| Missing | 2,129 (14.2) | 4,602 (3.9) | 36.5 |

^a^ Expressed as an absolute percentage

^b^ Asthma, chronic hypertension, insulin dependent diabetes, non-insulin dependent diabetes or heart disease

References

1. Dunn S, Bottomley J, Ali A, Walker M. 2008 Niday Perinatal Database quality audit: report of a quality assurance project. Chronic Dis Inj Can. 2011;32: 32–42.

2. Institute for Clinical Evaluative Sciences. Data Holdings [Internet]. Available: http://www.ices.on.ca/webpage.cfm?site_id=1&org_id=26&morg_id=0&gsec_id=5314&item_id=5322

3. Canadian Institute for Health Information. Data Quality Documentation, Discharge Abstract Database –– Multiyear Information. 2012.

4. Canadian Institute for Health Information. The Impact of the H1N1 Pandemic on Canadian Hospitals. 2010.
